# Rare subtypes of triple negative breast cancer: Current understanding and future directions

**DOI:** 10.1038/s41523-023-00554-x

**Published:** 2023-06-23

**Authors:** Alexandra Thomas, Jorge S. Reis-Filho, Charles E. Geyer, Hannah Y. Wen

**Affiliations:** 1 Department of Internal Medicine, Atrium Health Wake Forest Baptist Cancer Center, Winston-Salem, NC USA; 2 Department of Pathology and Laboratory Medicine, Memorial Sloan Kettering Cancer Center, New York, NY USA; 3 Department of Medicine, University of Pittsburgh UPMC Hillman Cancer Center, Pittsburgh, PA USA

**Keywords:** Breast cancer, Cancer epidemiology

## Abstract

Rare subtypes of triple-negative breast cancers (TNBC) are a heterogenous group of tumors, comprising 5–10% of all TNBCs. Despite accounting for an absolute number of cases in aggregate approaching that of other less common, but well studied solid tumors, rare subtypes of triple-negative disease remain understudied. Low prevalence, diagnostic challenges and overlapping diagnoses have hindered consistent categorization of these breast cancers. Here we review epidemiology, histology and clinical and molecular characteristics of metaplastic, triple-negative lobular, apocrine, adenoid cystic, secretory and high-grade neuroendocrine TNBCs. Medullary pattern invasive ductal carcinoma no special type, which until recently was a considered a distinct subtype, is also discussed. With this background, we review how applying biological principals often applied to study TNBC no special type could improve our understanding of rare TNBCs. These could include the utilization of targeted molecular approaches or disease agnostic tools such as tumor mutational burden or germline mutation-directed treatments. Burgeoning data also suggest that pathologic response to neoadjuvant therapy and circulating tumor DNA have value in understanding rare subtypes of TNBC. Finally, we discuss a framework for advancing disease-specific knowledge in this space. While the conduct of randomized trials in rare TNBC subtypes has been challenging, re-envisioning trial design and technologic tools may offer new opportunities. These include embedding rare TNBC subtypes in umbrella studies of rare tumors, retrospective review of contemporary trials, prospective identification of patients with rare TNBC subtypes entering on clinical trials and querying big data for outcomes of patients with rare breast tumors.

## Background

Triple-negative breast cancer (TNBC) constitutes an operational term describing a heterogenous group of tumors that are unified only by their shared lack of expression of estrogen receptor (ER) and progesterone receptor (PR), and lack of HER2 overexpression and/or *HER2* gene amplification. Molecular subtyping with gene expression profiles such as PAM50 have demonstrated the majority of TNBC cancers are of the basal subtype, which typify the biologically aggressive phenotype associated with TNBC. However, within this large group of TNBC tumors are an important subgroup of histologically rare tumors which display significant histologic and clinical heterogeneity. These rare TNBC tumors collectively represent a sizeable portion of TNBCs, accounting for 7% of TNBCs in one recent series^1^. Rare TNBCs carry a wide range of prognoses with a corresponding wide range of potential treatment options. The study of rare TNBCs has been confounded by factors including small numbers of cases, the use of overlapping or potentially related diagnoses, and variability in histologic interpretation. Contemporary tools, such as molecular classification of tumors, virtual pathology and big data, however, hold promise for advancing our knowledge about these understudied neoplasms. Here we review molecular and clinical features and treatment of more commonly encountered rare forms of TNBC, discuss how newer biomarker tools might facilitate disease management, and, finally, assess how contemporary research tools may provide disease-specific information for patients with these rare cancer types.

### Incidence of rare subtypes of TNBC

**Fig. 1:**
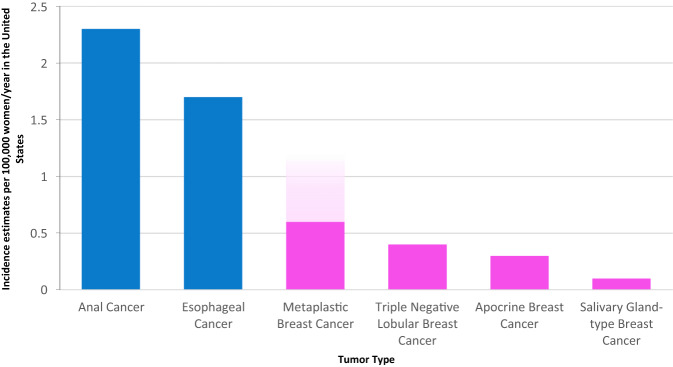
Incidence estimates of uncommon solid tumors and rare triple negative breast cancer subtypes. Solid tumors other than breast shown in blue. Rare subtypes of breast cancer shown in pink. Incidence is per 100,000 women/year in the United States (seer.cancer.gov/statfacts/)^4,44,76^. Light pink on top metaplastic breast cancer represents the possible extent of this tumor type which is likely underrepresented in databases due to diagnostic challenges and overlapping diagnosis codes^4,11,12^. (In comparison, the incidence of all breast cancer is 126.9/100,000 women/year in the United States.).

Rare cancers have been defined in a variety of ways, including the United States’ National Cancer Institute definition of 15 cases per 100,000 persons per year^2^, and the more conservative definition of 6 cases per 100,000 persons per year adopted by the European Union for Rare Cancer in Europe (RARECARE)^3^. These definitions provide a framework for the consideration of rare subtypes of TNBC tumors in the context of other less common solid tumors (Fig. 1). Individually, rare subtypes of TNBC are relatively uncommon; collectively, however, these tumors approach the incidence of other uncommon, but relatively well-studied solid tumors such as esophageal and anal cancers (1.7–2.3/100,000 women/year in the United States) (seer.cancer.gov/statfacts/ (accessed June 2, 2023)). In comparison, the incidence of all breast cancer is 126.9/100,000 women/year in the United States. When one adds other rarer TNBC subtypes, as well as hormone receptor positive subtypes such as mucinous and tubular breast cancers (both of which meet the RARECARE definition of rare), these rare tumors represent a sizeable portion of tumors encountered in clinical practice. Of note, the estimated incidences of rare breast tumors from large databases are likely underestimates, given that these tumors can be more difficult to diagnosis and may have a variety of categories into which they can be registered^4^. For example, diagnoses which would not have been included in the classic metaplastic breast cancer registration, include squamous cell carcinoma, spindle cell carcinoma, carcinosarcoma, pleomorphic carcinoma, to name a few possible synonyms.

### Review of rare subtypes of triple negative breast cancer

For the purpose of this review, rare subtypes of TNBC were selected from the 2019 World Health Organization (WHO) Classification of Tumors^5^. Tumors characterized by the expression of hormone receptors, or which are clinically ultra-rare fall outside the scope of this review and are not included.

### Invasive ductal carcinoma of no special type - medullary pattern

**Fig. 2:**
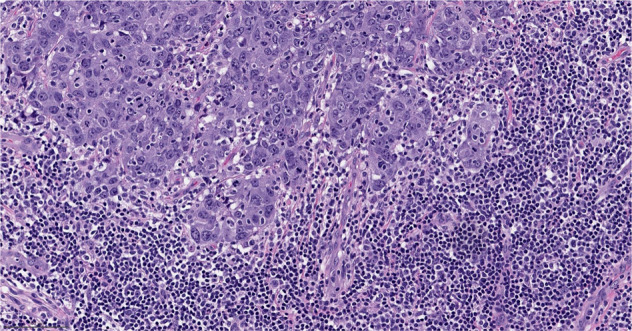
Invasive ductal carcinoma of no special type - medullary pattern. Morphologic features include high grade histology, syncytial architecture with no glandular structures, pushing margins, and prominent tumor infiltrating lymphocytes. (The term “syncytial growth” refers to a growth pattern characterized by broad, confluent bands of tumor cells more than five cells thick, often with indistinct cell borders^77^). Previously, described as “medullary carcinoma”, “atypical medullary carcinoma”, or “carcinoma with medullary features”, it is no longer classified as a special histologic subtype of invasive carcinoma according to the current WHO Classification. It is rather considered part of the spectrum of invasive carcinoma no special type, representing one end of the spectrum of the tumor infiltrating lymphocyte rich invasive carcinomas. H&E stain. Magnification 200x.

With the 5^th^ Edition of the WHO Classification, medullary carcinoma ceased to be considered a distinct subtype of breast cancer and was classified as a pattern within invasive ductal carcinoma of no special type^5^. Relatively high interobserver variability and overlap with basal-like tumors and breast cancers seen in patients with deleterious germline *BRCA1* pathogenic variants, led to the reassignment of this group of tumors in the most recent WHO classification^5,6^. This group of tumors is characterized by high grade features, dense lymphocytic infiltrate and are often well circumscribed (Fig. 2). Previous literature on this breast cancer subtype found that they have a favorable prognosis relative to invasive ductal carcinomas no special type^7^, and that they are responsive to chemotherapy^8^. These tumors are over-represented in the immunomodulatory subgroup of TNBCs^9^. Their excellent prognosis and responsiveness to therapy, relative to TNBCs of similar size receiving the same therapy, may place them in the group of tumors for which de-escalation studies may be considered^10^.

### Metaplastic carcinoma

**Fig. 3:**
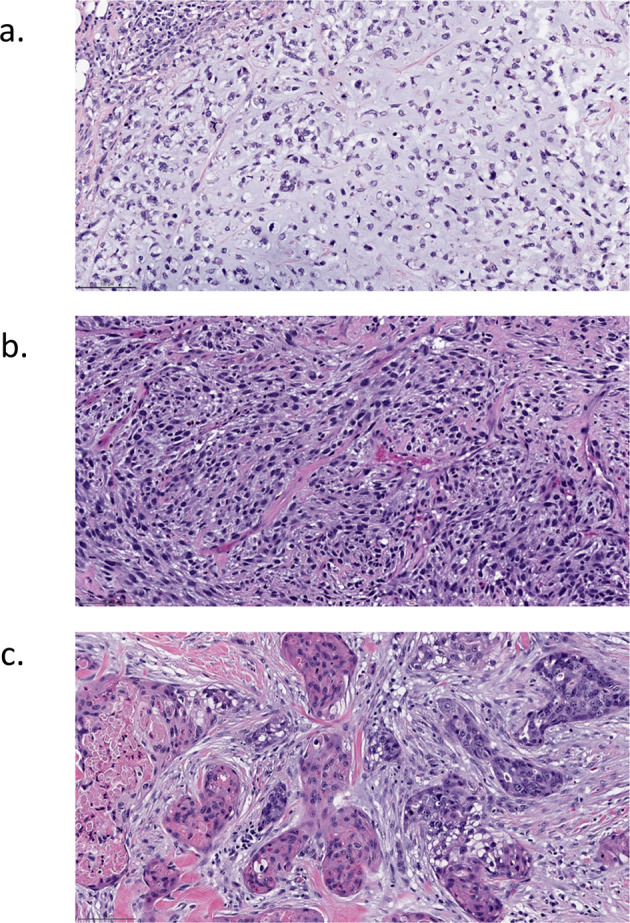
Metaplastic carcinoma. A heterogeneous group of invasive carcinomas characterized by squamous or mesenchymal differentiation. **a** Matrix producing subtype, with chondroid matrix. **b** Spindle cell carcinoma, with high grade spindle cells. The neoplastic cells are positive for high molecular weight cytokeratin by immunohistochemistry (not shown). **c** Squamous cell carcinoma, showing squamous differentiation. H&E stain. Magnification 200x.

Metaplastic breast cancers are a heterogenous group of invasive breast cancers which share differentiation toward squamous or mesenchymal-appearing elements (Fig. 3). The reported incidence of metaplastic breast cancer can vary from approximately 0.2% to 1.0% of breast cancers depending on the definition applied^11–13^. An analysis by intrinsic subtypes of 28 metaplastic breast tumors found the majority were claudin-low or basal-like intrinsic subtypes^14^. Among the triple-negative breast cancer subtypes, mesenchymal and basal-like were the most commonly identified subtypes in this series. The molecular subtype corresponded, to a large extent, with the histologic subtype analyzed in gene expression profiling studies, with tumors with a predominant spindle cell component uniformly classified in the claudin-low intrinsic subtype and tumors with a predominant chondroid component uniformly classified as the triple-negative mesenchymal subtype. Metaplastic breast cancers have a range of potential targets including DNA repair and alterations in the PIK3/AKT, MEK, and Wnt pathways. The frequency of these potential targets vary by the histologic component of the tumor, with squamous predominate tumors harboring an overrepresentation of PIK3CA alterations and some features suggestive of BRCAness, while spindle cell predominant tumors favor PIK3CA alterations and chondroid tumors appear more likely to harbor Wnt pathway alterations^15,16^. Metaplastic breast cancers tend to have higher PD-L1 expression than TNBCs of no special type and exhibit stem-like features which enable immune evasion and may also suggest vulnerability to immune therapy^17–20^.

Multiple series have reported inferior clinical outcomes for metaplastic carcinomas, which appear to have a propensity toward hematogenous spread rather than via the lymphatics, relative to TNBCs of no special type^11,21–23^. Further this tumor subtype demonstrates response rates to chemotherapy lower than typically seen with TNBC. Pathologic complete response (pCR) rates observed in single institution series of patients who received neoadjuvant therapy ranged from 2 to 11%^24–26^. Similarly, response rates reported with single agent chemotherapy for metaplastic tumors in the early and late line settings are lower than that typically seen for TNBC^27^. Although registry series have suggested improved outcomes in metaplastic breast cancer with chemotherapy relative to no chemotherapy and benefit with radiation relative to no radiation^28^, the low pCR rates in this subtype call into question the observation of an enrichment for genomic features of BRCAness in metaplastic breast cancers, given that one would expect higher rates of pCR in breast cancers with *de facto* homologous recombination DNA repair deficiency (HRD). Whole-genome sequencing studies and/or functional analysis of homologous recombination DNA repair are required to establish the prevalence of HRD in metaplastic breast cancers.

Exceptional responses to novel therapies in metaplastic breast cancer have been reported. The ARTEMIS trial recently reported on the experience of therapeutic escalation based on genomic characterization in patients with metaplastic breast cancer who had less than 70% decrease in tumor volume following neoadjuvant doxorubicin and cyclophosphamide^29^. In that trial, the pCR rate among the 39 patients with metaplastic tumors was 23%. A trial studying neoadjuvant therapy with single agent poly adenosine diphosphate-ribose polymerase (PARP) inhibition in patients with germline *BRCA* pathogenic variants reported an overall pCR rate of 53%, with 10 of 19 evaluable pateints achieving a pCR. In this trial, a single patient with metaplastic chondrosarcomatous breast carcinoma and a *BRCA2* germline pathogenic variant experienced a pCR following 6 months of treatment with single agent talazoparib^30^. In the advanced disease setting, there are reports of responses to checkpoint blockade, as well as to novel agents such as buparlisib, dabrafenib with trametinib and apatinib^31–34^. The DART trial offered a combination of nivolumab and ipilimumab to patients across a wide range of rare tumors, with each cohort comprised of a “basket” of patients with a specific rare tumor type. Cohort 36, comprised of patients with metaplastic breast cancer, completed accrual quickly with 3 of 17 patients experiencing long-term responses, though all three developed adrenal insufficiency^35^. Notably, responses were observed in tumors with low tumor mutational burden, low PD-L1 and absent TILs.

### Triple-negative invasive lobular carcinoma

**Fig. 4:**
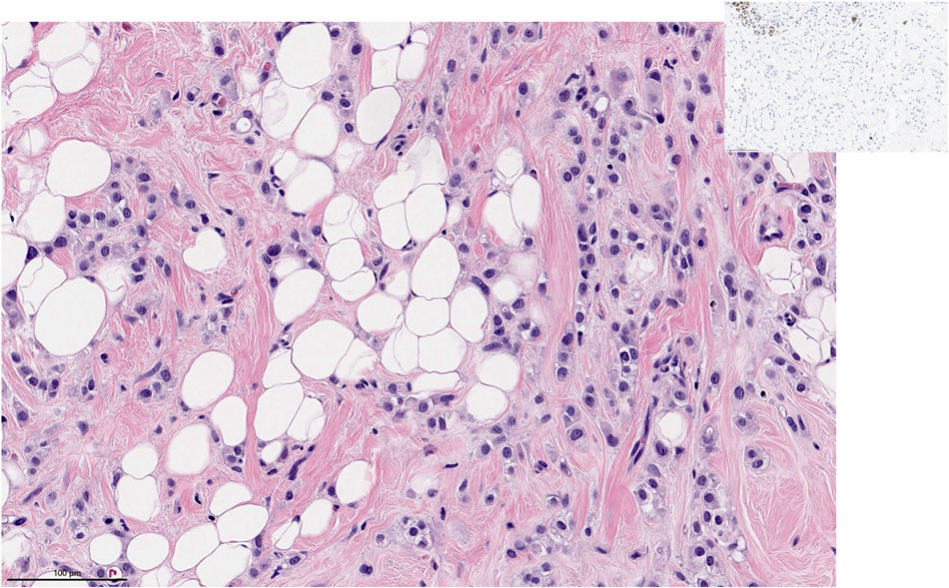
Triple negative invasive lobular carcinoma. Invasive lobular carcinoma, classic type, with discohesive neoplastic cells arranged in single file growth pattern invading the stroma. H&E stain. Magnification 200x. The picture insert demonstrates the absence of immunoreactivity for estrogen receptor (ER) in this tumor.

There is an increasing recognition of invasive triple-negative lobular carcinoma (ILC) as an uncommon manifestation of E-cadherin negative breast cancer. These tumors typically exhibit apocrine morphology, eosinophilic cytoplasm and prominent nucleoli (Fig. 4). Triple negative ILC accounts for 0.9–2.3% of invasive lobular breast cancers^36–39^. Patients with triple negative ILC also tended to be older than those in the comparator groups^40^. Reports on triple negative ILC are smaller, with numbers ranging in the dozens, though it appears that relative to patients with triple-negative invasive ductal carcinoma or hormone receptor positive ILC, patients with triple-negative ILC have inferior outcomes^36^. Others have also found that even though Ki67 tends to be lower in triple-negative ILC than in triple-negative invasive ductal carcinoma, outcomes are inferior for triple-negative ILC^40^.

**Fig. 5:**
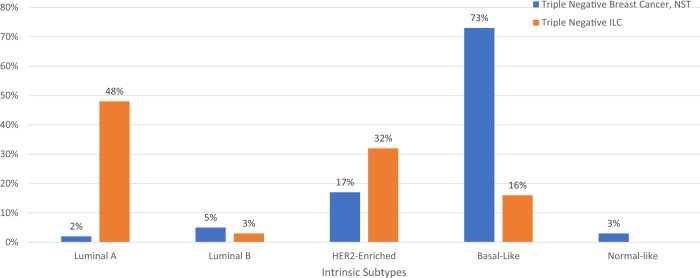
Breast cancer intrinsic subtypes in TNBC no special type and triple negative invasive lobular carcinoma. Portions of intrinsic molecular subtypes observed in TNBC no special type shown in blue^41^ and portions of subtypes observed in triple-negative invasive lobular carcinoma shown in orange^36^. TNBC Triple negative breast cancer, NST No special type, ILC Invasive lobular carcinoma, HER2 Human epidermal growth factor receptor 2.

A recent European series provided detailed molecular characterization of several dozen of these tumors; 23 of 31 (74%) expressed the androgen receptor, and 7 of 35 (20%) exhibited pathogenic human epidermal growth factor 2 (HER2) mutations^36^. These tumors are generally CK5/6 negative^40^. The proportion of intrinsic molecular subtypes represented in TNBC no special type and in triple negative ILC appear to differ, as seen in a comparison of a series which pooled 283 triple-negative tumors and the European series on triple negative ILC (Fig. 5)^36,41^. Triple negative ILC has relative overrepresentation of luminal A and HER2-enriched disease and is less likely to be basal-like^36^. The series also found that prognosis with triple negative ILC is superior for those with luminal-type disease relative to those with non-luminal like disease and that while the androgen receptor was expressed in many tumors, it was more likely to be expressed in luminal disease. *HER2* hotspot mutations were most commonly seen in tumors from the HER2-enriched subtype. Other potentially targetable features which over-represented in triple negative ILC included mutations of *PIK3CA* and DNA repair pathway alterations as well as higher tumor mutational burden^36^.

### Carcinoma with apocrine differentiation

**Fig. 6:**
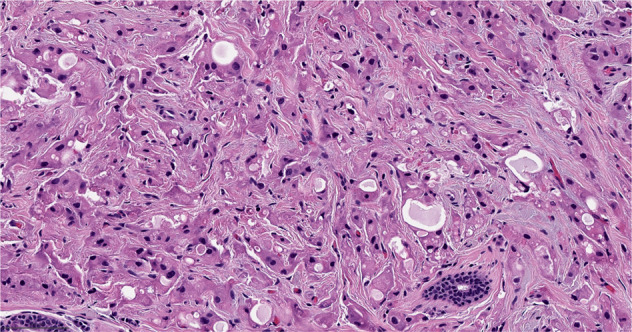
Carcinoma with apocrine differentiation. The neoplastic cells have abundant eosinophilic cytoplasm, enlarged nuclei with prominent nucleoli. The tumor cells are often positive for androgen receptor (AR). H&E stain. Magnification 200x.

This subtype of breast cancer is characterized by large cells with abundant eosinophilic granular cytoplasm and enlarged nuclei with prominent nucleoli, resembling apocrine sweat glands (Fig. 6). These tumors are frequently androgen receptor positive and exhibit *HER2* amplification in 30–60% of cases^5^. They tend to fall into the luminal androgen receptor and immune signature molecular subtypes of TNBC^9^, though a recent series noted they are often programmed death-ligand 1 (PD-L1) negative^42^. Most are sporadic, but these tumors are seen in patients with germline *PTEN* pathogenic variants^43^.

Relative to non-apocrine TNBC, patients with apocrine TNBC tend to be older and are more often white; these tumors also tend to be smaller and lower in grade^44^. While data on prognosis have been mixed, several series comparing triple-negative subtypes support a more favorable outcome for patients with apocrine carcinomas^44,45^. The magnitude of benefit from chemotherapy in these tumors is unclear, with mixed results from retrospective registries^1,46^. A smaller series reported some responses to neoadjuvant therapy in this tumor subtype^47^. These tumors are frequently of the luminal androgen receptor (LAR) subtype of triple negative breast cancer^9^, which has been shown to be associate with significantly lower pCR rates than other triple negative subtypes, in particular the basal-like subtype^48^. Responses to anti-androgen therapy in triple-negative androgen receptor positive breast cancer have also been reported^49,50^.

### Adenoid cystic carcinoma

**Fig. 7:**
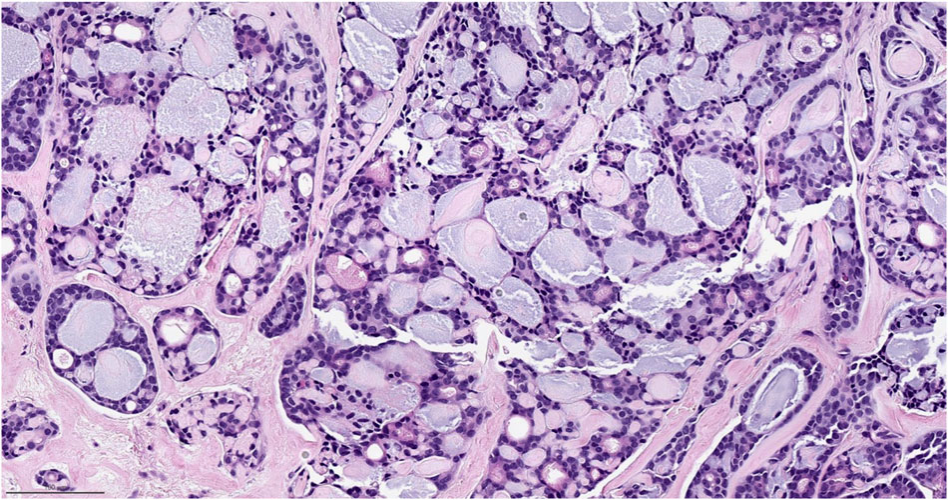
Adenoid cystic carcinoma. An example of classic adenoid cystic carcinoma, composed of epithelial and myoepithelial neoplastic cells, arranged in cribriform growth patterns. Pseudolumina are filled with basement membrane material. H&E stain. Magnification 200x.

Adenoid cystic carcinomas are a salivary gland-type tumor with low malignant potential composed of both myoepithelial and epithelial cells^51^. Classic adenoid cystic carcinoma has a cribriform pattern and a basophilic matrix (Fig. 7). The tumors commonly have *MYB-NFIB* fusions but can also have *MYBL1* rearrangements or *MYB* amplification^52^. Three subtypes have been described, classic adenoid cystic carcinoma, by far the most commonly diagnosed form of this subtype, and the less common, more aggressive subtypes: solid basaloid and high-grade transformational^51,53^. These tumors commonly present as a palpable mass in an older patient. Despite their triple-negative phenotype, prognosis for classic adenoid cystic carcinoma is excellent, and surgery is generally curative. Notably, tumors of similar histology and molecular phenotype arising in the salivary gland have a very different clinical behavior. Multiple retrospective series have shown no to marginal benefit from chemotherapy in these tumors^1,46^. The two less common subtypes, solid-basaloid and high-grade transformational adenoid cystic carcinoma, appear to have a more aggressive clinical course; and while numbers are small, benefit from chemotherapy in these subtypes cannot be ruled out^54^. Importantly, recent genomic analyses of solid-basaloid adenoid cystic carcinomas demonstrated that only a minority of these cancers harbor the cardinal molecular features of classic adenoid cystic carcinomas, suggesting that taxonomically, these solid-basaloid carcinomas likely constitute a convergent phenotype, with only a small subset being pathogenetically related to adenoid cystic carcinomas^53^.

### Secretory carcinoma

**Fig. 8:**
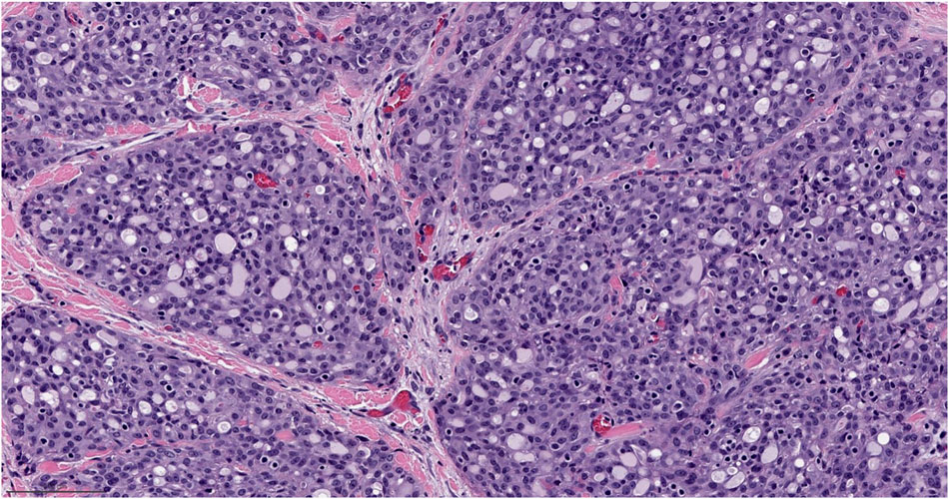
Secretory carcinoma. The tumor cells have abundant intracytoplasmic vacuoles and extracellular secretions. H&E stain. Magnification 200x.

Secretory breast carcinoma has features quite distinct from those commonly seen in in breast oncology. These tumors often exhibit the presence of large amounts of intracellular and extracellular secretions and can resemble thyroid follicles (Fig. 8). Importantly, they exhibit a pathognomonic *ETV6-NTRK* fusion gene^55,56^. This gene encodes a tyrosine kinase which activates RAS-MAPK and PI3K pathways to drive oncologic processes. TRK inhibitors can be associated with profound and long-lasting responses and at least one case of a dramatic response to larotrectinib in advanced pediatric secretory breast carcinoma has been reported^57^.

These tumors present as slow growing, often painless mobile masses and can be associated with nipple discharge^58^. Mean age at diagnosis was 56 years in a recent series^59^, though these tumors can also occur in children. These are triple-negative tumors with an excellent prognosis which can generally by managed by local therapies alone^59^. While rare, distant metastases can occur, and in this context, TRK inhibitors should be considered.

### Neuroendocrine carcinoma

**Fig. 9:**
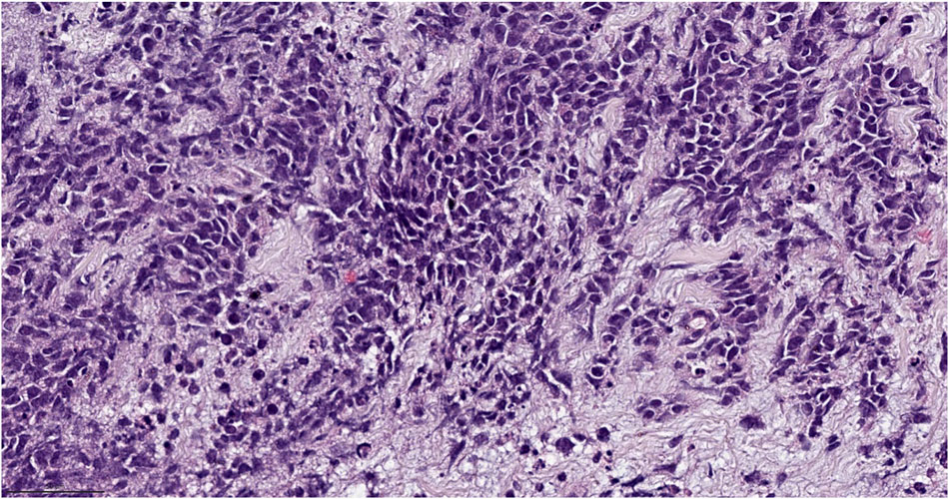
Small cell carcinoma. High grade carcinoma exhibiting neuroendocrine morphology with hyperchromatic cells, high N:C ratio, and scant cytoplasm. Immunohistochemical stains show the tumor cells are positive for cytokeratin, neuroendocrine markers (synaptophysin, chromogranin), negative for TTF-1, with high Ki67 proliferation index (>90%) and loss of RB protein expression (not shown). Magnification 200x.

Neuroendocrine carcinoma of the breast is most frequently a small cell carcinoma, which represent 3–10% of extrapulmonary small cell carcinomas^60,61^. Neuroendocrine carcinomas of the breast tend to be poorly differentiated and hormone receptor negative. In contrast, neuroendocrine tumors of the breast are generally well differentiated and hormone receptor positive. Importantly, neuroendocrine tumors of the breast are not equivalent to carcinoid tumors at other disease sites. In the breast, the predictive and prognostic implications of neuroendocrine features are less clear, thus adjuvant treatment strategies used for invasive breast cancer no special type are also utilized for neuroendocrine breast tumors^5,62^. The distinct forms of neuroendocrine neoplasia of the breast, lower grade tumors versus high grade carcinomas, may develop from different pathways, similar to what has been reported for pancreatic neuroendocrine neoplasia^63,64^. To be identified as a pure neuroendocrine carcinoma greater than 90% neuroendocrine component is required^5^. When these carcinomas originate in the breast, the most common manifestation is that of an extrapulmonary small cell, though less common large cell neuroendocrine carcinomas can occur. These carcinomas are characterized by high-grade neuroendocrine morphology supported by the presence of neurosecretory granules (Fig. 9). These carcinomas are frequently characterized by *TP53* and *RB1* alterations^63^. *PIK3CA* mutations have been reported, though the clinical implications of this are less certain^65^.

In neuroendocrine carcinoma it is critical to confirm that the breast lesion is not a metastasis from another primary site. The presence of ductal carcinoma in situ or invasive breast carcinoma no special type supports breast origin. Extrapulmonary small cell carcinoma of the breast is more likely to present as limited disease than other extrapulmonary sites of small cell carcinomas^60^. Survival with local and regional disease is superior to that of stage matched patients with small cell lung carcinoma^60,66^. Patients with primary small cell carcinoma of the breast may be eligible for small cell lung cancer trials, typically earlier phase trials (e.g., ClinicalTrial.gov Identifier NCT03896503).

### Harnessing contemporary biologic tools to better understand rare subtypes of TNBC

In special subtypes of TNBC limited disease-specific information is available which generally makes the path toward individualized therapy less clear than in TNBC no special type. Disease molecular features suggest possible vulnerability to targeted approaches (Table 1), though the clinical value of these is known to be variable and often of limited degree. Generally, the higher risk tumors may be candidates for therapy escalation and the lower risk tumors for de-escalation, though exceptions exist in either direction. Importantly, individual patients may have disease which allows treatment with subtype agnostic therapies such as checkpoint blockade with PD-L1 positivity or high tumor mutational burden^67^ or PARP inhibition with germline BRCA pathogenic variants^68^.

**Table 1:**
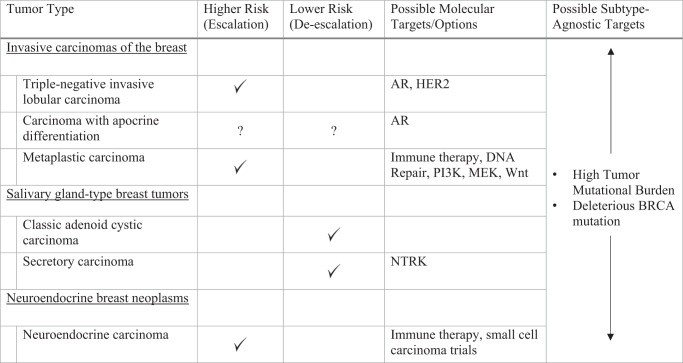
Possible molecular therapeutic approaches in uncommon subtypes of triple negative breast cancer.

Data are emerging that biomarkers used in TNBC no special type and breast oncology more broadly will likely have utility in rare triple-negative subtypes. The ARTEMIS trial demonstrated pCR correlated with improved survival in metaplastic breast cancer and that on therapy imaging can assess the efficacy of neoadjuvant therapy which could support imaging as a biomarker of response and limit exposure to inactive chemotherapy^29^.

Circulating biomarkers are rapidly emerging as tools for noninvasive real-time monitoring of the tumor course and response to therapy. While these will intuitively apply to rare tumors, emerging case reports are providing support for this approach. A case report of a women with small cell carcinoma of the cervix found circulating tumor DNA levels tracked with initial response to therapy and disease recurrence^69^. In metaplastic spindle cell breast carcinoma, a patient with disease refractory to chemotherapy and bevacizumab had a marked response to apatinib and the tumor mutational profile tracked with these changes^33^. Additionally, patients with uncommon TNBC tumors may be eligible for trials studying circulating biomarkers, for example patients with metaplastic breast cancers and triple negative ILCs would not typically be excluded from trials of TNBC. Prospective or retrospective review to identify and study patients with these tumors could confirm disease-specific utility of biomarker tools.

### Harnessing other tools for disease-specific answers on rare subtypes of TNBC

Currently, the information available on rare subtypes of TNBC comes mostly from retrospective reviews of large databases, as well as from small case series, and individual case reports. Albeit helpful, these sources all have limitations, though an array of approaches, which utilize established and newer tools, hold promise in advancing disease-specific understanding in rare breast cancer subtypes (Table 2). Individual clinical trials across the spectrum of rare TNBC tumors would be costly and impractical to conduct. While randomized trials offer greater scientific validity if successfully completed, they are impractical in rare diseases. It can be cost and resource prohibitive for individual sites to open and operate trials which target rare patient populations. A success in this space has been the series of phase II trials conducted in rare gynecologic tumors by NRG (GOG) though most trials have been single arm and have required international collaboration^70^.

**Table 2:** Possible opportunities to improve disease-specific understanding of rare breast cancer subtypes.

Opportunity Category	Examples
Clinical Trials	Prospective disease-specific trials
	Basket trials enrolling multiple cohorts, each with a distinct rare tumor (e.g.: DART)
	Retrospective review of outcomes for patients with rare tumors enrolled on completed trials
	Prospective identification of patients with rare tumors for new trials or on-going platform trials
Infrastructure	International collaborations (e.g., International Rare Cancers Initiative)
	Engage cooperative groups (e.g., ETCTN) which enrich for mega-centers where patients with rare breast cancer subtypes often receive care
Technology	Real world data to provide retrospective information or to intentionally follow a cohort of patients with a rare subtype of breast cancer prospectively
	Digital pathology and artificial intelligence to prospectively review and register tumors to reduce issues related to overlapping diagnoses and interobserver variability

Novel approaches to trial design and prospective identification of rare breast cancer subtypes in participants entering trials have been used to build understanding of rare tumors. The DART trial successfully embedded an uncommon breast cancer subtype in a larger basket trial of rare tumors^35^. Metaplastic breast cancers were prospectively identified 39 of the 211 participants enrolled in ARTEMIS and has provided disease-specific information^29^. Harnessing contemporary tools such as digital pathology could make such prospective identification more facile than in earlier eras.

‘Big data’, the use of large, variable and complex datasets, offers another contemporary approach, which could advance our understanding of rare subtypes of TNBC^71^. The FDA has noted that real world data have applications in rare diseases, when clinical trials are impractical^72^. An example of how such information can help address questions in less common breast cancer populations includes the effort to utilize real world data to support a label expansion for palbociclib in men^73^. Big data could also offer systematic insight into ultra-rare breast tumors. Finally, other emerging technologic tools, digital pathology and artificial intelligence, could hold the potential to facilitate more accurate registration of rare tumor subtypes, thereby limiting registration issues associated with overlapping diagnostic groups and interobserver variability^74,75^.

## Discussion

Collectively, rare subtypes of TNBC are encountered in clinical practice with some frequency and a better understanding of these subtypes could improve outcomes and minimize overtreatment. We have some, albeit limited, disease-specific information on how to treat special subtypes (e.g., secretory carcinomas). As biologic principles applied to TNBC no special type extend to less common subtypes we can improve our understanding of this group of breast tumors. While clinical trial information would be helpful, emerging technologies may offer additional, more practical tools, to accrue sufficient subtype specific data to deliver on the promise of personalized cancer care for patients with rare forms of TNBC.

### Reporting summary

Further information on research design is available in the Nature Research Reporting Summary linked to this article.

## Supplementary information

Reporting Summary
